# Late-Onset, Autosomal Dominant, Axonal, Sensorimotor Neuropathy Due to the New Variant c.1A_G in Myelin Protein Zero (MPZ): A Case Report

**DOI:** 10.7759/cureus.90315

**Published:** 2025-08-17

**Authors:** Josef Finsterer

**Affiliations:** 1 Neurology, Neurology and Neurophysiology Center, Vienna, AUT

**Keywords:** autosomal dominant, axonal neuropathy, mpz gene, nerve conduction studies, sensorimotor

## Abstract

To the best of our knowledge, autosomal dominant Charcot-Marie-Tooth disease type 1B (CMT1B) due to the c.1A>G variant in myelin protein zero (MPZ) has not yet been reported.

The patient was a 56-year-old man with slowly progressive atrophy of the distal muscles of the lower limbs that started in his forties. He later developed transient numbness in both feet. One year before presentation, he noticed paresthesia in the distal lower limbs and easy fatigability when walking uphill. His family history revealed hereditary neuropathy in his mother and her two sisters. Genetic testing of his mother and an aunt revealed the new variant c.1A>G in MPZ (p.Met1Val). Clinical neurological examination revealed myopia, hypoacusis, absent tendon reflexes on the upper and lower limbs, mild weakening of the left thumb and right foot extensors, hypoesthesia and hypoalgesia on both soles and dorsum of the feet, and mild pallhypesthesia on the lower limbs. Nerve conduction studies (NCS) revealed severe sensorimotor axonal neuropathy. The index patient also carried the mutation identified in his mother and aunt.

The MPZ variant c.1A>G manifests phenotypically with late-onset CMT1B. Neurologists should be aware that hereditary neuropathy can have a late onset and a slowly progressive course over a number of years, with the ability to walk unimpaired into old age.

## Introduction

Hereditary neuropathies belong to a large group of disorders of the peripheral nervous system (PNS) that are caused by mutations in more than 100 genes and manifest as motor, sensory, autonomic, or multisystemic abnormalities. They are inherited in an autosomal dominant, autosomal recessive, X-linked, or maternal manner [1,2]. Hereditary neuropathies are currently classified based on a combination of genetic data, inheritance patterns, nerve conduction studies (NCS), and phenotype [1,2]. There are four main groups, including hereditary sensorimotor neuropathies (HSMN), hereditary sensory neuropathies (HSN), hereditary motor neuropathies (HMN), and hereditary sensory and autonomic neuropathies (HSAN) [2]. Clinically, they manifest as numbness, tingling, pain in the feet or hands, muscle weakness, muscle wasting, and, when autonomic fibers are affected, sweating disorders, orthostatic hypotension, and insensitivity to pain [2]. Some patients may have foot deformities, thin calf muscles, locking similar to that of an upside-down champagne glass, or scoliosis.

One of the genes that is frequently mutated in hereditary neuropathies is the myelin protein zero (MPZ) or P0 gene [3]. The MPZ gene, located on 1q23.3, encodes the myelin protein zero, a membrane glycoprotein and important component of the myelin sheath in peripheral nerves [3]. The MPZ gene consists of at least one promoter (P0 promoter) and six exons. MPZ is involved in cell-cell adhesion and myelination via plasma membrane adhesion molecules. The MPZ protein is mainly expressed by Schwann cells and accounts for more than 50% of the proteins in the PNS [3]. It has also been found to be expressed in Rohon-Beard neurons, the basal plate region of the midbrain, the central nervous system, the cranial nerves, and oligodendrocytes [4]. MPZ mutations cause myelin deficiency and are responsible for Charcot-Marie-Tooth disease 1B (CMT1B) (OMIM 118200), CMT2I/J, and hypomyelinating or dysmyelinating neuropathy (Dejerine-Sottas disease) [1]. The prevalence of CMT1B, the most common MPZ-related neuropathy, is estimated to be approximately one in 30,000 individuals [5]. MPZ mutations account for an estimated 4% to 5% of all CMT patients [5]. To the best of our knowledge, an autosomal dominant CMT1B due to the c.1A>G variant has not yet been described.

## Case presentation

**Figure 1:**
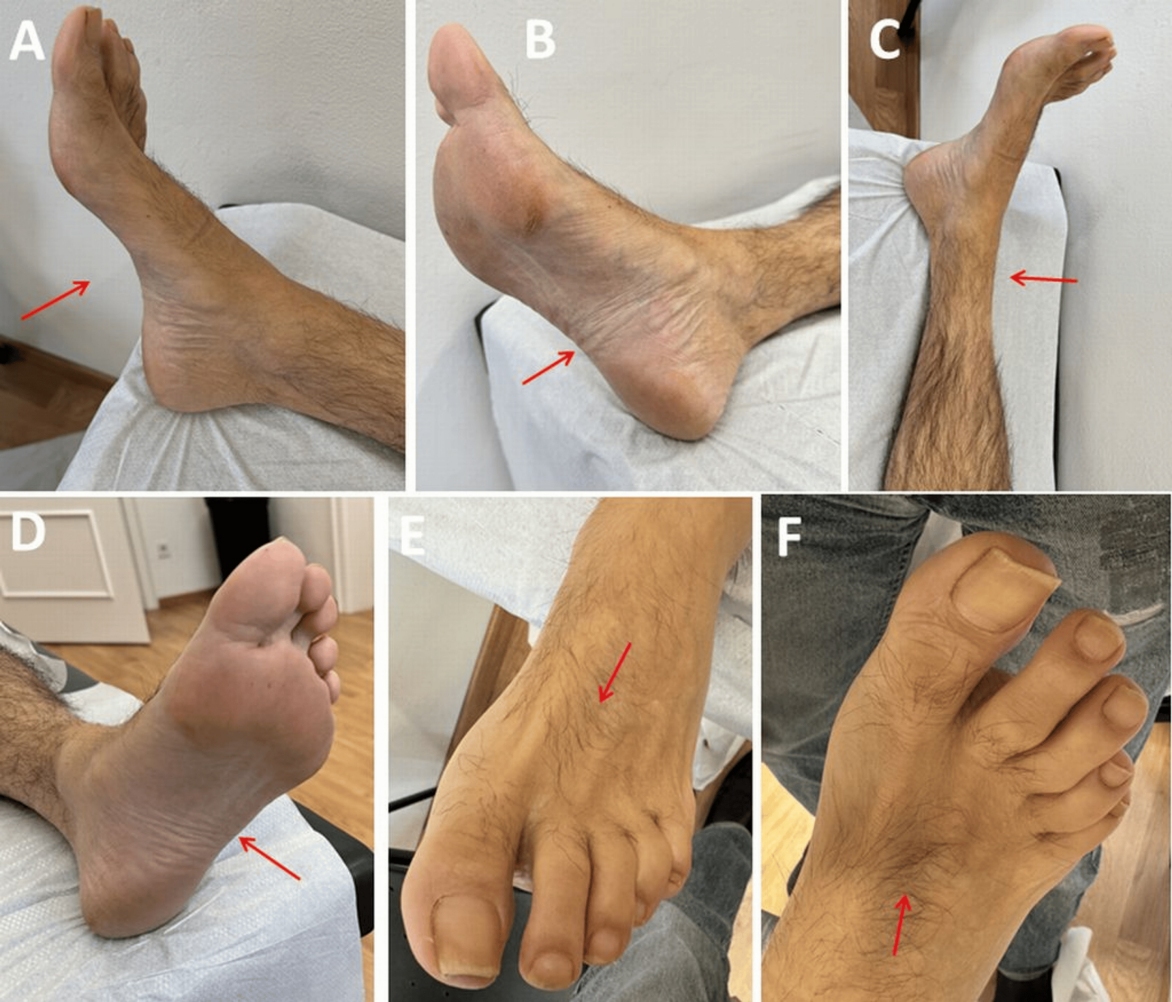
Right (panels 1-3, panel 6) and left (panels 4-5) lower limbs of the index patient with muscle atrophy of the intrinsic foot muscles and distal lower leg muscles, as well as slight foot deformity (pes cavus), There was no trophic disorder.

The patient was a 56-year-old man with autosomal dominant axonal hereditary neuropathy of the CMT1B type due to the heterozygous variant c.1A>G in MPZ. He presented with slowly progressive muscle weakness in the distal lower extremities, which began in his early forties (Figure 1). Later, he developed numbness in both feet. For about a year, he complained of paresthesia in the distal lower extremities, which was more severe at night than in the morning. He noticed slight fatigue, especially when walking uphill. His medical history also included exhaustion and depression at the age of 32, for which he had to be hospitalized twice for psychiatric treatment. Since then, he has received high-dose neuroleptic and antidepressant treatment with duloxetine (120 mg/day), quetiapine (600 mg/day), prothipendyl (160 mg/day), and clonazepam (2 mg/day). At the age of 54, he underwent a prostatectomy for prostate cancer without the need for radiation or chemotherapy. At the age of 55, he fell 3 meters onto his back without sustaining any major injuries or disabilities.

**Figure 2:**
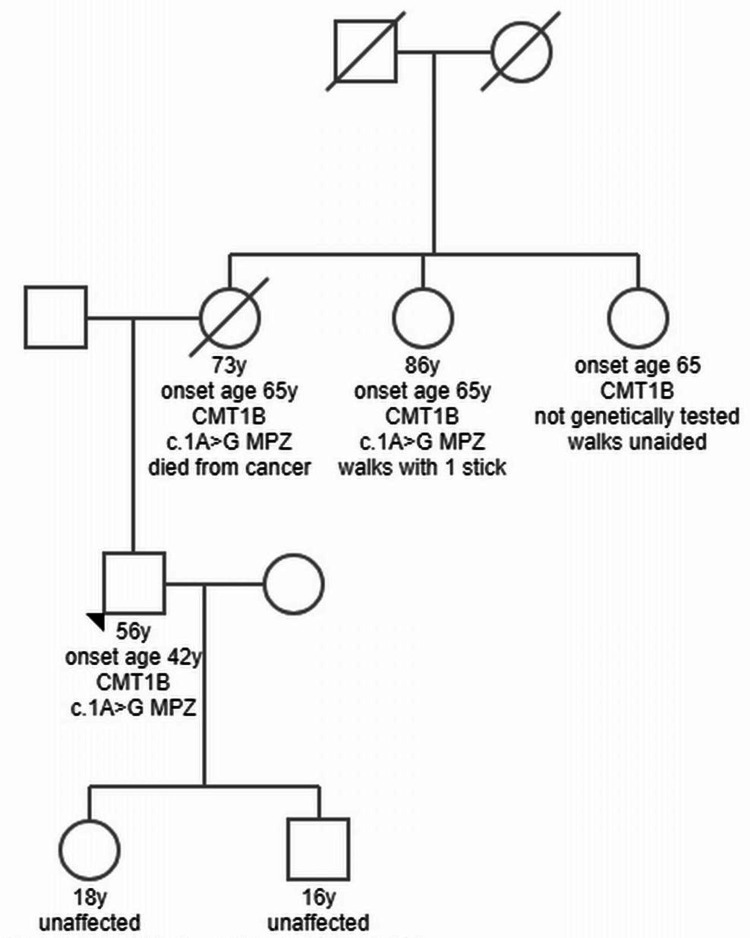
Pedigree of the index patient CMT1B: Charcot-Marie-Tooth disease type 1B; MPZ: myelin protein zero

His family history included cases of hereditary neuropathy in his mother and her two sisters. The symptoms in his mother and aunts appeared at the earliest at the age of 65 and initially affected only the hands. His mother had since died of esophageal cancer, but one aunt was still able to walk independently, while the other required a walking aid. His two teenage children had not yet been clinically affected (Figure 2). Genetic testing of his mother and one of his aunts revealed the c.1A>G variant in MPZ (p.Met1Val), which was responsible for the neuropathy in these two patients.

**Figure 3:**
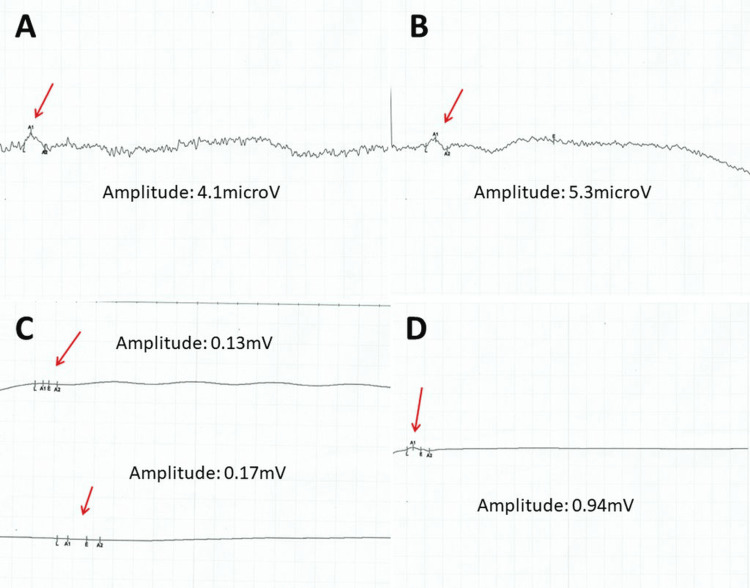
Results of nerve conduction studies of the right median nerve (panel A, sensory), left median nerve (panel B, sensory), left peroneal nerve (panel C, motor), and left tibial nerve (panel D, motor)

Clinical neurological examination revealed myopia, mild hearing loss, absent tendon reflexes in the upper extremities, mild muscle atrophy of the left thenar, mild weakness of the right foot extension (M5-), hypesthesia and hypoalgesia on both soles and dorsum of the feet, mild pallhypaesthesia in the lower extremities, and absent tendon reflexes in the lower extremities. The knee-hook attempt was restricted on the right side. NCS at the age of 56 revealed severe sensorimotor axonal neuropathy of the median, tibial, and peroneal nerves (Table 1), predominantly in the lower extremities (Figure 3). Genetic testing of the MPZ variant found in his mother and aunt confirmed that the index patient was also a carrier of this variant. Causes of secondary neuropathy due to metabolic, endocrine, toxic, infectious, inflammatory, paraneoplastic, or neoplastic diseases were thoroughly excluded. He was advised to take pregabalin or gabapentin, but he refused.

**Table 1:** Results of nerve conduction studies na: not available, nm: not measurable

Nerve	Distal latency (ms)	Amplitude	Velocity (m/s)
Right median motor	4.3	7.35mV	38
Right median sensory	na	4.1mV	47
Left median motor	4.0	2.95mV	38
Left median sensory	na	5.9mV	45
Right tibial motor	6.2	0.15mV	34
Left tibial motor	6.0	0.94nV	nm
Right peroneal motor	nm	nm	nm
Left peroneal motor	10.1	0.17mV	nm
Right sural	nm	nm	nm
Left sural	nm	nm	nm

## Discussion

The patient presented here is interesting in several respects. First, he carried the new variant c.1A>G in the MPZ gene, which manifested phenotypically as late-onset, autosomal dominant, sensorimotor, axonal length-dependent neuropathy. This particular variant has not yet been described as a cause of CMT1B. The c.1A>G variant was classified as pathogenic because it was responsible for CMT1B in at least three family members, because its phenotypic expression was similar to that observed in patients with other MPZ mutations [6], and because the variant resulted in an amino acid change from methionine to valine. The causality of the MPZ variant was also suggested by the fact that extensive investigations into alternative causes of sensorimotor neuropathy did not reveal a causal etiology. Second, this variant may be associated with a type of anticipation, as the index patient developed the disease phenotypically about 10 years earlier than his mother and aunts. In addition, his disease began in the legs, while his mother's disease began in the upper limbs. Third, contrary to previous reports [7,8], the patient did not complain of pain. It has been reported that up to 80% of patients with MPZ variants experience pain [3]. The index patient, therefore, did not require medication for neuropathic pain, dysesthesia, or allodynia. The reason why the patient did not show any pain remains unknown, but it can be speculated that the causative mutation is a loss-of-function mutation leading to a severe deficiency or absence of the functional MPZ protein (null allele or haploinsufficiency). It is also conceivable that modifier genes suppressed the severity of the disease by acting on the same biological pathway as the disease-causing gene. It can also be assumed that axonal defects in CMT1B cause less or no pain compared to demyelinating defects.

The previously reported genotypic spectrum of patients with MPZ mutations included more than 200 mutations [9]. MPZ mutations cause misfolding and retention of MPZ in the sarcoplasmic reticulum of myelinating Schwann cells. The molecular consequences of the c.1A>G mutation identified in the index patient are unknown, as no studies on the functional consequences have been conducted. However, MPZ mutations appear to act via gain-of-function mechanisms [10], but MPZ glycosylation also appears to play a key role in the pathophysiology [10]. A gain of function through glycosylation is more harmful to MPZ transport and functionality than a loss of function through glycosylation [10]. The spectrum of mutations varies between regions [11]. Mutations in MPZ are usually inherited in an autosomal dominant manner [12].

The phenotypic spectrum of MPZ variants encompasses not only large fiber neuropathy but is more widespread. MPZ can manifest with isolated small fiber neuropathy [7], not only with demyelinating but also with axonal neuropathy [13], hearing loss [14], respiratory muscle weakness [15], tremor, ataxia, cognitive impairment, and arrhythmias [15]. Late onset of CMT1B has been reported previously [16,17] and may not be related solely to the type of underlying mutation, as complete loss of MPZ can also be associated with late onset and a phenotype similar to that of the index patient [17]. Although hearing loss was only mild in the index patient, it was attributed to the underlying MPZ variant, as other MPZ mutations have previously been described that manifest as hearing loss and hearing impairment [14,18]. In general, CMT1B with late onset is usually mild and slowly progressive [19,20]. As far as diagnosis is concerned, a nerve biopsy is not necessary for CMT1B, as it can only yield nonspecific findings but no pathognomonic features [3].

## Conclusions

In summary, this case demonstrates that the MPZ variant c.1A>G manifests phenotypically as late-onset CMT1B and that this new variant expands the genotypic spectrum of MPZ mutations. Neurologists should be aware that hereditary neuropathy due to MPZ mutations can occur late in life, that successive generations may have an earlier onset, and that the course may be slowly progressive over many years, allowing these patients to walk independently into old age. Hereditary neuropathy should be suspected when there is neuropathy, hearing impairment, and foot deformity. Patients with suspected hereditary neuropathy should undergo genetic testing once all causes of secondary (acquired) neuropathies have been thoroughly ruled out. Nerve biopsy plays a minor role in the diagnosis of hereditary neuropathies.

## References

[1] Hayes LH, Sadjadi R (2023). Hereditary neuropathies. Continuum (Minneap Minn).

[2] Rossor AM, Evans MR, Reilly MM (2015). A practical approach to the genetic neuropathies. Pract Neurol.

[3] Bertini A, Gentile L, Cavallaro T (2024). Phenotypic spectrum of myelin protein zero-related neuropathies: a large cohort study from five mutation clusters across Italy. J Neurol Neurosurg Psychiatry.

[4] (2025). Charcot-Marie-Tooth disease, demyelinating, type 1b; cmt1b. https://omim.org/entry/118200.

[5] Bremer J, Meinhardt A, Katona I (2024). Myelin protein zero mutation-related hereditary neuropathies: neuropathological insight from a new nerve biopsy cohort. Brain Pathol.

[6] McCulloch MK, Mehryab F, Rashnonejad A (2024). Navigating the landscape of CMT1B: understanding genetic pathways, disease models, and potential therapeutic approaches. Int J Mol Sci.

[7] Gemignani F, Percesepe A, Gualandi F (2024). Charcot-Marie-Tooth disease with myelin protein zero mutation presenting as painful, predominant small-fiber neuropathy. Int J Mol Sci.

[8] Stojkovic T, de Seze J, Dubourg O, Arne-Bes MC, Tardieu S, Hache JC, Vermersch P (2003). Autonomic and respiratory dysfunction in Charcot-Marie-Tooth disease due to Thr124Met mutation in the myelin protein zero gene. Clin Neurophysiol.

[9] Zhu J, Guo G, Mehryab F (2025). Generation of two induced pluripotent stem cell lines from Charcot-Marie-Tooth type 1B patients harboring autosomal dominant mutations in myelin protein zero gene. Stem Cell Res.

[10] Veneri FA, Prada V, Mastrangelo R (2022). A novel mouse model of CMT1B identifies hyperglycosylation as a new pathogenetic mechanism. Hum Mol Genet.

[11] Taniguchi T, Ando M, Okamoto Y (2021). Genetic spectrum of Charcot-Marie-Tooth disease associated with myelin protein zero gene variants in Japan. Clin Genet.

[12] Hayasaka K, Himoro M, Sato W (1993). Charcot-Marie-Tooth neuropathy type 1B is associated with mutations of the myelin P0 gene. Nat Genet.

[13] Iyer VG, Shields LB, Zhang YP, Shields CB (2023). Clinical features of a newly described mutation of myelin protein zero in a family. Cureus.

[14] Duan X, Gu W, Hao Y (2016). A novel Asp121asn mutation of myelin protein zero is associated with late-onset axonal Charcot-Marie-Tooth disease, hearing loss and pupil abnormalities. Front Aging Neurosci.

[15] Mandich P, Fossa P, Capponi S (2009). Clinical features and molecular modelling of novel MPZ mutations in demyelinating and axonal neuropathies. Eur J Hum Genet.

[16] O'Connor G, McNamara P, Bradley D, Connolly S, Langan Y, Redmond J (2012). Late-onset CMT phenotype caused by a novel mutation in the MPZ gene. Eur J Neurol.

[17] Gharesouran J, Hosseinzadeh H, Naghiloo A (2023). Complete loss of myelin protein zero (MPZ) in a patient with a late onset Charcot-Marie-Tooth (CMT). Metab Brain Dis.

[18] Seeman P, Mazanec R, Huehne K, Suslíková P, Keller O, Rautenstrauss B (2004). Hearing loss as the first feature of late-onset axonal CMT disease due to a novel P0 mutation. Neurology.

[19] Kleffner I, Schirmacher A, Gess B, Boentert M, Young P (2010). Four novel mutations of the myelin protein zero gene presenting as a mild and late-onset polyneuropathy. J Neurol.

[20] Pisciotta C, Bertini A, Tramacere I (2023). Clinical spectrum and frequency of Charcot-Marie-Tooth disease in Italy: data from the National CMT Registry. Eur J Neurol.

